# Dead Teeth in the Jaws

**Published:** 1885-10

**Authors:** Truman W. Brophy


ARTICLE III.

### DEAD TEETH IN THE JAWS.

TRUMAN W. BROPHY, M. D., D. D. S.

In reply to Dr. Sexton on this subject, Dr. Brophy makes these pertinent remarks in the journal of the American Medical Association:

Dr. Sexton says: "The retention in the jaws of teeth which are diseased, have become irredeemably sensitive to thermal influences, or deprived of adequate periosteal nourishment through calcareous formations about the roots, very frequently gives rise to nervous diseases about the head. I am convinced that these reflected nerve influences manifest themselves much oftener since dentistry has come more extensively into practice during the present generation. and greater efforts are made to retain defective teeth in the jaw."

That diseases of the teeth are often the center from which pain is reflected to the eyes, ears and other parts, all experienced clinical observers must admit. But that these pathological conditions of the teeth, from which reflected pain has its origin, can be and are successfully treated and cured with rare exceptions, as effectually as any other diseases, is a fact too well established to be set aside.

It is not possible to describe in this letter the method by which the various diseases of the teeth are treated, but suffice it to say that "teeth which are diseased from death of the pulp or from caries" *do not* "become irredeemably sensitive to thermal influences." In proof of this statement, many thoroughly educated medical men, practicing the specialty of dental surgery, will testify.

"Teeth deprived of adequate pereosteal nourishment, through calcareous formations about the roots, very frequently give rise to nervous diseases about the head." To this statement I assent, but dissent as to the remedy not mentioned but implied. *i. e.*, the removal of the teeth. If the calcareous deposits mentioned have destroyed so much of the pericementum and the alveolar processes as to render the teeth very loose; if, indeed, the teeth have lost their bony support and are retained by means of a remnant of pericementum only, they cannot, of course, be restored to permanent health and usefulness, and their removal is, therefore, indicated. Teeth in this condition "frequently give rise to nervous diseases about the head."

On the contrary, if the calcareous depostits have not destroyed the pericementum and alveolar processes to a very great extent, the condition is amendable to intelligent treatment and cure. In answer to the assertion that "Reflected nerve influences manifest themselves much oftener since dentistry has come more extensively into practice during the present generation," I would say, that with equal propriety it might be said that reflected nerve influences manifest themselves more frequently since gynæcology has come more extensively into practice. To attribute the obvious increase of nervous diseases during the present generation to diseases of the teeth is a statement not only "sweeping," but "overdrawn." *Much harm* is no doubt done by some of the modern appliances "for retention in the mouth of substitutes for absent teeth," and the unhealthy state of the gums and contiguous parts, established and maintained by the presence of these substitutes, unquestionably give rise in many cases to reflected pain.

When Dr. Sexton attempts to establish a *law* governing the management of diseased teeth, it must be based on more substantial grounds than those which he presents. The case related of his patient, the "medical man, who practices dentistry," and who was convinced that an inflammation of one of his ears began from the time the upper second molar of that side was treated for a diseased pulp, is simply an assumption, on the part of the patient, that the ear trouble had its origin from the diseased tooth, and the patient's diagnosis of his own case seems to have been accepted by Dr. S. as conclusive. The ear disease in this case may have emanated from the diseased tooth, but no evidence is produced to that effect. In regard to the query as to "whether it is safe practice to retain dead teeth in the jaws," I would say that thousands of people in our own country have had pulpless (not dead) teeth in their jaws many years, which are exempt from pericemental disease, and which serve all the purposes for which teeth were provided. To ask whether it is safe practice to retain these, so-called, dead teeth in the jaws when they have been comfortable and useful from ten to forty years and promise to remain so through life, seems like a proposition too injudicious to need comment. While the death of the pulp results in "cutting off the source of nutrition from the dentine," it does not follow "that in a large number of instances irritation can not be easily controlled."

Neither does the tooth become a foreign substance. The dentine and the enamel are, of course, no longer nourished after the death of the pulp, but their resisting structure renders them capable of maintaining their integrity many years after the pulp has been removed; and pericementum will nourish the cementum and thereby retain the tooth in its alveolus in a comfortable condition. In order, however, to thus retain the tooth and prevent inflammation from supervening, the devitalized pulp must be removed, the pulp canals thoroughly disinfected and filled with a plastic material which hardens when in position. Dr. S. most clearly exhibits his imperfect knowledge of the dental operations in vogue when he says: "Inflammation of exposed dentine cannot surely be entirely arrested in any case by filling the pulp cavity with any known extraneous material, and especially is handicraft wanting to even imperfectly protect the minute and often tortuous canals leading down to the apical foramina of the majority of the teeth." To arrest "inflammation of exposed dentine by filling the pulp cavity," in the opinion of Dr. S. would seem to be most desirable. How a tissue without nourishment and consequently without vitality can take or maintain inflammation is beyond comprehension. The impervious filling which I have mentioned will close the apical foramina, together with the canal, which "in the majority of cases" *is not* tortuous to a degree of rendering the perfect filling of the root difficult or uncertain, and the assertion that the dental surgeon "is able only to offer a hopeful but uncertain prognosis in these cases" is contrary to well established fact. There are no diseases to which mankind is heir more scientifically and effectually cured than the diseases of the teeth in question.

Again: "The dead tissues of the dentine will sooner or later, most likely, be transmitted through the tissues of the cementum to the periosteum." Communication between the lacunæ canaliculi of the cementum with the tubuli of the dentine is not free; indeed, it seldom exists, hence it cannot be "that through the periosteum alone the dentine may long derive some nourishment."

About 22,000,000 teeth are annually extracted in the United States, and I regret to say this enormous loss of teeth is to no small extent due to the indifference manifested by physicians in the anatomy, physiology and pathology of these organs. It is a fact, no one will attempt to gainsay, that hygienic measures directed toward the preservation of the deciduous set, if understood, are seldom recommended by the general practitioner to the families under his charge. The premature loss of these teeth paves the way for early lesions of the permanent set. The pain resulting from advanced caries of the deciduous teeth, owing to the difficulties encountered in controlling the patient, is not easily treated; moreover, the injurious impressions thus made on the system of the child abide through life. There is no doubt hundreds of thousands of teeth are unnecessarily extracted each year, and then drugs are given with a view of curing the patient of the disorders of digestion and other abnormal conditions which follow, and which in turn arise from imperfect mastication of food, verily for the want of teeth.

We need to know "what's the matter" in the treatment of these "nervous diseases about the head," as in all others, and apply a remedy which will bring the abnormal tissues back to health. Too often, indeed, has it happened that patients, by advice of their medical attendants, have submitted to the loss of many, and, in some instances, to all their teeth, in the vain endeavor to be relieved from trigeminal neuralgia. You may ask, Why this useless loss of teeth, and all the resulting evils? Because the advice given was not wise; the etiology of the affection was not understood.

There are certain pathological conditions of the teeth which have not been mentioned in this discussion, and which give rise to reflected pain of the eyes, ears, and other parts.

Among these may be mentioned exostosis of the roots of teeth and nodules of calcific matter within the pulp canals in contact with a living pulp. The former of these conditions has been regarded incurable, the removal of the tooth with the united bony tumor being indicated. In favorable cases, however, this tumor may be excised and removed without removing the tooth. The pulp nodules of calcified deposits within the pulp chamber may be, in a large majority of cases, successfully removed without sacrificing the tooth.

No one approves more than I the removal of the causes of disease. It is no more necessary to extract a tooth at the root of which an alveolar abscess has formed than it would be to amputate a limb for the cure of an abscess of the medullary substance of its bone. Disease of the eye sometimes requires that it be enucleated, but the honest, skilled ophthalmologist *would not* remove the eye when he *knew* he could restore it to usefulness. The spirit of the teachings of Dr. Sexton's articles is far from being progressive. Nor is this all; many assertions are not based on fact, but on erroneous impressions. Our duty to our profession and the laity is not to destroy but to save; and while ignorance is ever working its mischief in all vocations in life, it is not just to accept the results of such work as a basis on which to found a law.

